# Genome-Wide Association Study of Chlorophyll Fluorescence and Hyperspectral Indices in Drought-Stressed Young Plants in Maize

**DOI:** 10.3390/genes16091068

**Published:** 2025-09-11

**Authors:** Lovro Vukadinović, Vlatko Galić, Maja Mazur, Antun Jambrović, Domagoj Šimić

**Affiliations:** Agricultural Institute Osijek, 31000 Osijek, Croatia; lovro.vukadinovic@poljinos.hr (L.V.); vlatko.galic@poljinos.hr (V.G.); maja.mazur@poljinos.hr (M.M.); antun.jambrovic@poljinos.hr (A.J.)

**Keywords:** crops, water withholding, leaf spectroscopy, genetic dissection, SNP markers

## Abstract

Background/Objectives: Global maize production is considerably affected by drought aggravated by climate change. No genome-wide association study (GWAS) or candidate gene analysis has been performed using chlorophyll fluorescence (ChlF) and hyperspectral (HS) indices measured in young plants challenged by a water deficit. Our objective was to conduct a GWAS of nine ChlF and HS indices measured in a diversity panel of drought-stressed young plants grown in a controlled environment using a maize single nucleotide polymorphism (SNP) 50k chip. Methods: A total of 165 inbred lines were genotyped using the Infinium Maize50K SNP array and association mapping was carried out using a mixed linear model. Results: The GWAS detected 37 respective SNP markers significantly associated with the maximum quantum yield of the primary photochemistry of a dark-adapted leaf (Phi_Po), the probability that a trapped exciton moves an electron into the electron transport chain further than QA (Psi_o), the normalized difference vegetation index (NDVI), the Zarco–Tejada and Miller Index (ZMI), greenness, modified chlorophyll absorption in reflectance (MCARI), modified chlorophyll absorption in reflectance 1 (MCARI1), and Gitelson and Merzlyak indices 1 and 2 (GM1 and GM2). Conclusions: Our results contribute to a better understanding of the genetic dissection of the ChlF and HS indices, which is directly or indirectly related to physiological processes in maize, supporting the use of HS imaging in the context of maize breeding.

## 1. Introduction

Maize (*Zea mays* L.) is a globally important crop due to its versatility and importance in food and feed, through which it contributes to global food security, animal agriculture, and various industrial sectors. However, drought exacerbated by climate change significantly impacts maize production, reducing yields and affecting various growth stages [1]. Early development stages, including germination, emergence, root development, and early leaf production reaching the 6–7 leaf stage, are critical for determining the final plant population density and setting the stage for future development. Under extreme conditions during this period, water deficit can result in complete seedling establishment failure [2].

Maize generally exhibits some resilience to drought, and recent research has focused on developing drought-tolerant varieties and implementing management strategies to mitigate the effects of drought. It is crucial to determine which agronomic and other traits are associated with drought tolerance [3]. Apart from agronomic or physiological traits such as water use or photosynthetic efficiency, there is a need to determine additional secondary phenotypic traits [3].

Recently developed high-throughput phenotyping methods could be a novel tool for estimating agronomically relevant traits [4], leveraging recent progress in conventional, molecular, and transgenic breeding for drought resilience in maize. These methods encompass a multitude of optical sensors and methods that capture different electromagnetic responses at different wavelengths [4]. In controllable environments, high-throughput plant phenotyping includes, among other methods, chlorophyll fluorescence (ChlF) and hyperspectral (HS) imaging [5], obtaining useful data about the physiological state of the crop in a fast and noninvasive manner. Use of ChlF in maize research on drought tolerance is well documented globally [6,7,8,9]. The same applies for HS imaging data [10,11,12]; however, data on the use of ChlF and HS imaging for maize germplasm screening remain limited [13].

Researchers use molecular markers associated with yield and other drought-related traits in genomics studies to identify genes and quantitative trait loci (QTLs) for drought tolerance. Sheoran et al. [14] stated that drought stress tolerance in maize could be enhanced by combining (gen)omics with high-throughput phenotyping. In their review, reference lists of QTL analyses and genome-wide association studies (GWASs) for drought tolerance traits in maize were given. However, no study is cited in which ChlF or HS parameters were used, although it was emphasized that combining omics technologies and high-throughput phenotyping should contribute to disentangling the molecular and physiological bases of drought tolerance features. Nevertheless, the HS parameter normalized difference vegetation index (NDVI) [15] was used in a comprehensive GWAS aiming to genetically dissect seasonal dynamics through aerial-based high-throughput phenotyping in field environments with no specific stress [16].

Recently, high-throughput phenotyping with HS parameters (indices) was used to conduct a GWAS and candidate gene analysis in durum wheat grown under heat and drought stress [17]. Jin et al. [18] conducted a GWAS to identify SNP markers (single-nucleotide polymorphism) associated with chlorophyll content in maize during the whole vegetation period, but no ChlF or HS parameters were used. Similarly, a GWAS and candidate gene analysis for chlorophyll content in fifth leaves at the early stage and in ear leaves at the filling stage were presented [19]. A GWAS for some physiological stress-related traits was performed in a diversity panel of elite maize inbred lines challenged by withholding water [2], but no ChlF or HS measurements were carried out.

As far as we know, no GWAS or candidate gene analysis has been carried out using ChlF and HS indices measured at the early plant stage in a maize germplasm collection challenged by drought stress. The objective of this study was to conduct a GWAS of nine ChlF and HS indices measured in drought-stressed young plants of a maize diversity panel grown in a controlled environment using a maize SNP 50k chip.

## 2. Materials and Methods

### 2.1. Plant Materials

The diversity panel comprised 165 maize inbred lines previously described by Vukadinović et al. (2025) [13], wherein the respective genetic structure was presented in detail. Most of the lines are elite material coming from the maize breeding programs of Agricultural Institute Osijek located in Southeastern Europe (European Corn Belt). The origin of this material is mostly US Corn Belt germplasm. Population structure was determined through Admixture analysis [20], which detected seven maize groups, namely, sweet corn, Lancaster, Ohio, flint/pop, B73, B37, and Iodent, whereby 101 were admixed lines, 20 were Iodent, 15 were B73, 15 were Ohio, and 14 were of Lancaster origin. The inbred lines were genotyped using an Illumina Infinium Maize50K array (Illumina Inc., San Diego, CA, USA) [21]. The final genomic dataset was constructed by filtering the whole diversity panel data to maximum 5% missing data, minor allele frequency of 0.02, and no heterozygotes. Filtering resulted in 33,001 sites that were imputed by using LinkImpute methodology [22] with 30 sites in high linkage disequilibrium and 10 nearest neighbors.

### 2.2. Phenotyping

The phenotyping procedure and methods are described in detail elsewhere [13]. In brief, maize plants were grown in a climate chamber FITOCLIMA 12000 PLH (Aralab, Lisbon, Portugal) for 28 days from planting to the V6 stage (establishment phase). The growing conditions were 16 h of daylight with a temperature of 25 °C and relative humidity of 65% and 8 h of darkness (night) with a temperature of 18 °C and relative humidity of 90%.

Six cycles were required for the complete set of maize inbreds, which underwent two simultaneous treatments: control and water withholding. The control group was watered daily, and the water withholding treatment involved withholding water from the 10th day after planting (no watering). Measurements of ChlF and HS imaging of the leaves were conducted at three different time points: Measurement 1 was performed on the 14th day after planting, Measurement 2 was performed on the 20th, and Measurement 3 was performed on the 26th day after planting. The same six plants of every genotype were measured at every time point, presenting six technical replicates per genotype (an inbred line) in each of the two treatments (control and water withholding) in each measurement.

Three hand-held devices were used, including MP100 for ChlF parameters utilizing the JIP-test [23], PolyPen RP 410 NIR (near-infrared spectra) measuring in the 640–1050 nm range, and PolyPen RP 410 UVIS (ultraviolet and visible spectra) measuring hyperspectral response in the 380–790 nm range. All devices were from PhotonSystem Instruments (PSI, Drásov, Czech Republic). Compared to data for 15 indices presented in a previous study [13], the results presented herein are shown for nine indices in which significant associations with specific SNPs were detected. The indices were divided into three groups: ChlF, NIR, and UVIS. The ChlF indices were the maximum quantum yield of the primary photochemistry of a dark-adapted leaf (Phi_Po) [23] and the probability that a trapped exciton moves an electron into the electron transport chain further than QA (Psi_o) [23]. The NIR indices were normalized difference vegetation (NDVI) [24] and the Zarco–Tejada and Miller index (ZMI) [25]. The UVIS indices were greenness [26], Gitelson and Merzlyak index 1 (GM1) [27], Gitelson and Merzlyak index 2 (GM2) [27], the modified chlorophyll absorption in reflectance index (MCARI) [28], and modified chlorophyll absorption in reflectance index 1 (MCARI1) [29].

### 2.3. GWAS and Canidate Genes

Phenotypic data were mean-aggregated within each combination of measurement treatment and genotype and combined with genomic data for association mapping (AM). AM was carried out in Tassel software version 5.2.96 [30,31] using the mixed linear model procedure, accounting for population structure (Q), as assessed by means of principal coordinate analysis with six axes, and kinship (K) calculated using the identity-by-state method with up to six ancestral alleles, i.e., MLM + Q + K. The Bonferroni threshold for declaring the significance of association was determined following the simpleM procedure described in [32,33]. Briefly, of the 33,001 filtered and imputed markers, the effective number of markers (M_eff_) was determined to be 8201 and the significance threshold of α = 0.05 was divided by the M_eff_, which resulted in a –log(*p*) value of 5.214.

The most significant SNP was selected to represent the locus associated with ChlF and HS indices in the same LD block. The annotated genes were searched within the 100 kb region around (50 kb upstream and 50 kb downstream) the detected significant SNP using the Zm-B73-REFERENCE-NAM-5.0 database. Gene names refer to Ensembl BioMart [34] and description and GO domains refer to MaizeGDB [35]. Only the protein-coding genes were analyzed.

## 3. Results

In Table 1, means, standard deviations, and minimum and maximum values together with coefficients of variation are presented for nine ChlF and HS indices in six treatment/measurement combinations each. Generally, there were differences between the two treatments for all indices. As expected, standard deviations were larger in water withholding than in the control, which became even larger during the course of the experiment. The differences among three measurements in a particular treatment were greater in the water withholding treatment, although some differences also existed among three measurements in the control carried out at three different early development stages. Out of 54 treatment/measurement combinations across the traits, only 15 significant genome-wide associations were detected.

**Figure 1:**
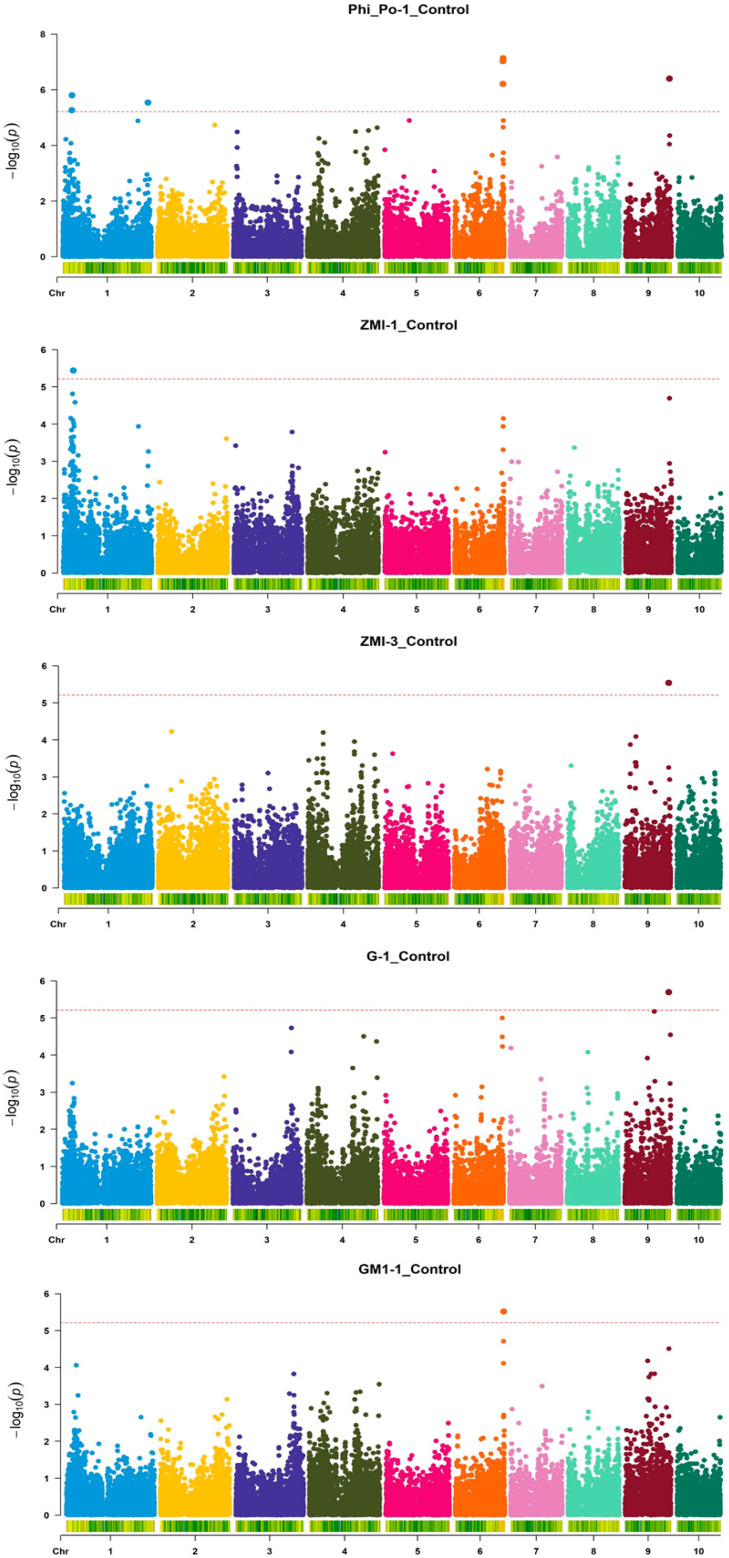
Manhattan plots of the GWAS of four ChlF or HS indices (Phi_Po, ZMI, Greenness (G), and GM1) in young maize plants for five Index-Measurement_Treatment combinations. The dotted lines indicate the genome-wide significance threshold –log(*p*) value of 5.214 (α = 0.05).

**Figure 2:**
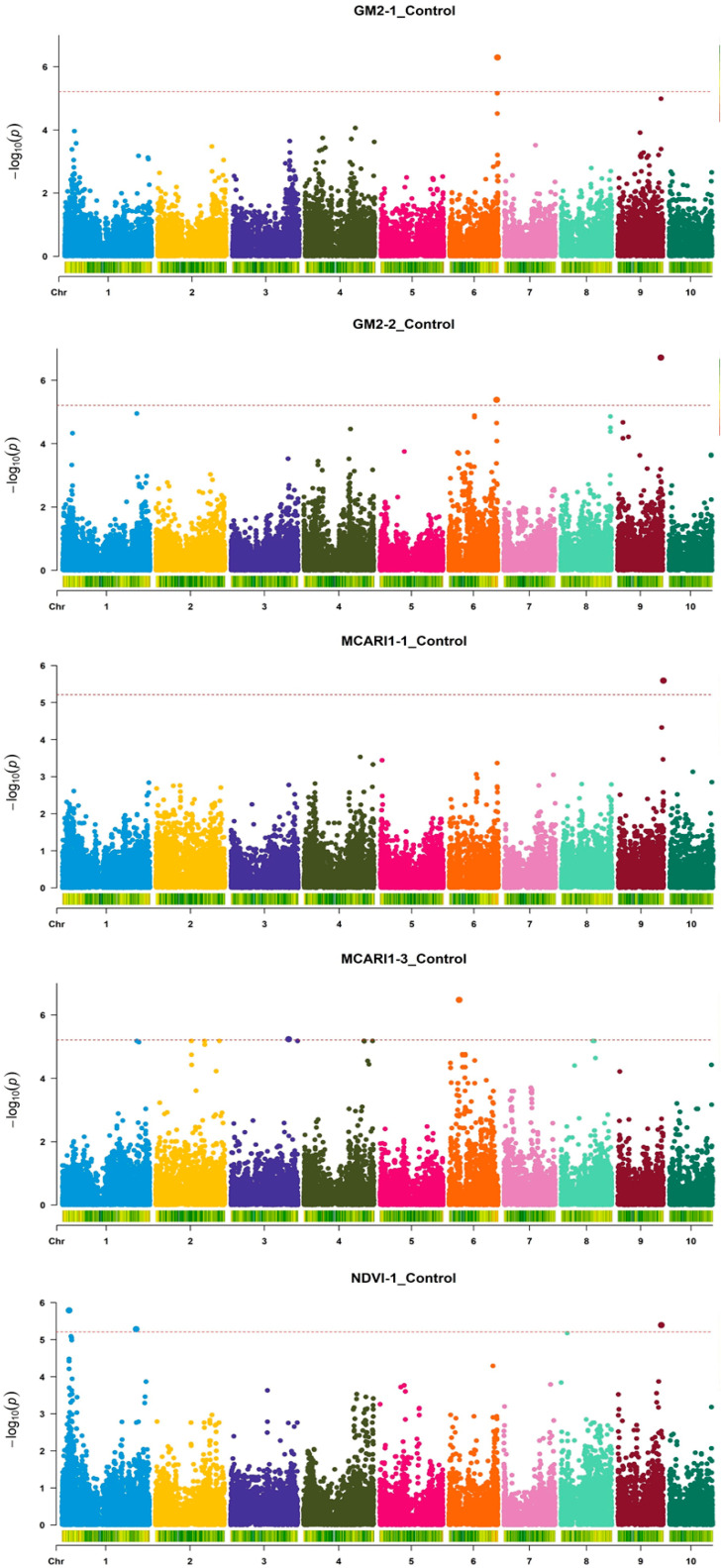
Manhattan plots of the GWAS of three HS indices (GM2, MCARI1, and NDVI) in young maize plants for five Index-Measurement_Treatment combinations. The dotted lines indicate the genome-wide significance threshold –log(*p*) value of 5.214 (α = 0.05).

**Figure 3:**
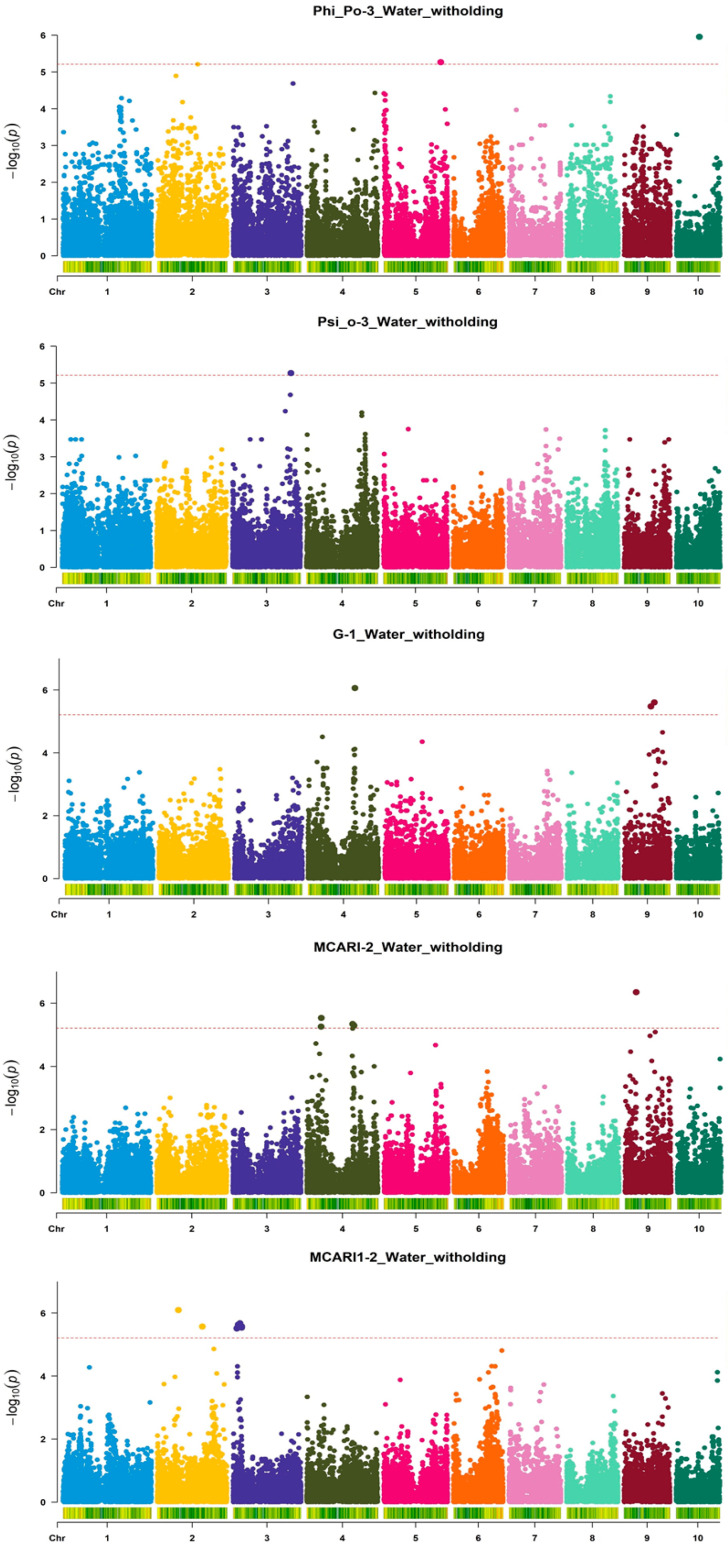
Manhattan plots of the GWAS of five ChlF and HS indices (Phi_Po, Psi-o, Greenness (G), MCARI, and MCARI1) in young maize plants for five Index-Measurement_Treatment combinations. The dotted lines indicate the genome-wide significance threshold –log(*p*) value of 5.214 (α = 0.05).

Altogether, 37 SNPs across eight chromosomes were significantly associated with respective indices in 15 measurement/treatment combinations (Figure 1, Figure 2 and Figure 3): nine each for Phi_Po and MCARI1; five for MCARI; four for greenness; three for GM2 and the NDVI, respectively; two for ZMI; and one for Psi_o and GM1, respectively.

In the control, seven significant SNPs were detected for Phi_Po in Measurement 1 on chromosomes 1, 6, and 9, as well as per one SNP for ZMI (Measurements 1 and 2) and greenness and GM1 in Measurement 1 (Figure 1). Also in the control, one SNP was detected for GM2 and MCARI1, respectively, both in Measurement 1; two SNPs for GM2 were detected in Measurement 1 and for MCARI1 in Measurement 3, respectively, as well as three SNPs for the NDVI in Measurement 1 (Figure 2). These SNPs were positioned on chromosomes 1, 3, 6, and 9. In water withholding, one SNP was detected for Psi_o in Measurement 3 on chromosome 3; two SNPs for Phi_Po were also detected in Measurement 3 (chromosomes 5 and 10); three SNPs for greenness were detected in Measurement 1 (chromosomes 4 and 9); five SNPs for MCARI were detected in Measurement 1 (also chromosomes 4 and 9); and six SNPs for MCARI1 were detected in Measurement 2 (chromosomes 2 and 3) (Figure 3).

Among the 37 significant associations between SNPs and indices in all measurement/treatment combinations, 5 of them had no candidate genes match in the Ensembl BioMart database [34]. The exact chromosomal positions of the other 32 SNPs on their respective chromosomes and candidate genes significantly associated with the indices are presented in Table 2, Table 3 and Table 4, where a description of the genes and their gene ontology (GO) domains are also shown. Altogether, 61 candidate genes were found. However, many of them had no obvious biological relation with the traits that they were statistically associated with. On chromosome 1, eight SNPs were in the vicinity of 18 coding-protein candidate genes. The SNP PZE-101206617 was associated with two indices (Phi_Po and ZMI) both measured in the control in Measurement 1. For this, there is one candidate gene coding Gibberellin-regulated protein 1 (Table 2). On Chromosomes 2, 3, and 4, twelve significant SNPs were related to fifteen candidate genes (Table 3). Two SNPs, SYN571 and SYN572, associated with MCARI1 in water withholding in Measurement 1 were both related to one candidate gene coding an uncharacterized protein. On chromosomes 5 and 6, seven SNPs were related to sixteen candidate genes (Table 4). Two SNPs SYN4313 and SYN4314 had four common candidate genes associated with three indices (Phi_Po, GM1 and GM2) all measured in thecontrol in Measurement 1. Here, two genes were described as protein kinase domain-containing protein genes. The SNP SYN4302 had the same candidate genes, but it was associated only with Phi_Po. On chromosomes 9 and 10, there were five SNPs significantly associated with indices related to 12 candidate genes (Table 5). The SNP PZE-109031829 was associated with ZMI measured in the control in Measurement 3 and MCARI measured in water withholding in Measurement 3. PZE-109108465 was associated with five indices, Phi_Po, greenness, GM2, MCARI1, and the NDVI, all measured in the control. The majority of the candidate genes had all three GO domains of molecular function, cellular component, and biological process.

## 4. Discussion

Our previous study showed that there is considerable variability within the diversity panel for the 15 ChlF and HS indices [13]. However, for some of them, no significant associations with SNPs were detected. This is particularly true for the performance index on an absorption basis (Pi_Abs), as a prominent ChlF index for which several QTLs were previously detected in different maize populations [36,37]. On the other hand, several significant associations were found for a maximum quantum yield of primary photochemistry of a dark-adapted leaf (Phi_Po or Fv/Fm) on chromosomes 1, 5, 6, 9, and 10. For this parameter, a significant QTL was previously detected at a normal growing temperature (control) of 25 °C, also on chromosome 6, by evaluating cold stress [38]. Additionally, several QTLs were found on a few chromosomes [39] when the QTLs for leaf stay-green-associated parameters were analyzed. GWAS signals associated with variation in a number of spatially corrected photosynthetic traits including Phi_Po were identified in the field environment with no specific stress [40]. A GWAS identified 205 significant marker–trait associations for a number of chlorophyll fluorescence parameters, including Phi_Po in wheat grown under different sowing conditions [41].

Notably, a group of NIR indices had no significant associations, whereas all five UVIS indices were associated with a number of SNPs detected either in the control or water withholding groups. In ten combinations, significant associations with SNPs in the control environment were detected, while in half of them (5), associations were detected in water withholding. In total, 20 significant SNPs related to protein-coding genes were detected in the control, whereas only 12 were detected in water withholding. Candidate gene analysis revealed that the SNPs identified in the control group were consistently collocated with candidate genes related to proteins that are ubiquitously present in plants performing diverse developmental and physiological functions during the maize life cycle in non-stress environments (e.g., protein kinase domain-containing protein). On the other hand, in water withholding, genes that play a role in stress environments were identified (e.g., transcription factor MYB36, protein detoxification—multidrug and toxic compound extrusion protein). The only conflicting significant SNP is PZE-109031829 on chromosome 9, simultaneously associated with ZMI in the control and MCARI in water withholding.

There were also direct associations between an index and the SNP/candidate gene that takes part in the photosynthetic process, including the NDVI in the control group with SYN30050 (rubredoxin-like domain-containing protein; pet7—photosynthetic electron transport7) with the GO terms electron transport activity, chloroplast, and electron transport chain and MCARI in the water withholding group in Measurement 2 (STI1 domain-containing protein and Hsp70-Hsp90 organizing protein 3 with the GO terms thylakoid and plastid). The NDVI was also used in a comprehensive GWAS to genetically dissect seasonal dynamics through aerial-based high-throughput phenotyping in field environments with no specific stress [16].

A GWAS and candidate gene analysis in durum wheat grown under heat and drought stress showed not only significant SNP associations with MCARI and NDVI indices [17] but also physical positions for marker trait associations that are common for several HS indices. The same was true in our study, wherein SNPs PZE-101206617, SYN4313, and PZE-109031829 on chromosomes 1, 6, and 9, respectively, were associated with two ChlF and/or HS indices (Table 2, Table 4 and Table 5). Moreover, PZE-109108465 was associated with five indices, i.e., one ChlF index (Phi_Po) and four HS indices based on ultraviolet and visible spectra (greenness, GM2, MCARI1, and NDVI) measured in the control.

Using ChlF and HS imaging, maize breeders can rapidly screen large numbers of maize genotypes for improved stress resistance by also providing in-season predictions of performance [42]. In combination with association studies, this can eventually contribute to the faster development of drought-tolerant and/or high-yielding hybrids. However, because our investigation used a smaller sample size, some of the associations might be suggestive. All significant SNPs did reach rigorous statistical significance, but further research with larger sample sizes is needed to determine if they are true markers or merely statistical anomalies. The next step should be to conduct a GWAS on the indices using a larger sample size of maize inbred lines from Southeast Europe. In a comprehensive analysis of 572 inbreds from this region, a number of selection signatures have been detected and scanned for candidate genes [43] after an analysis of more the 460,000 molecular markers via a 600 k Maize SNP Genotyping Array. In total, 101 genes were associated with the GO term response to stimulus, of which the majority were associated with response to stress. However, no phenotyping including ChlF and HS imaging was carried out in that study.

## 5. Conclusions

The use of ChlF and HS radiometry as high-throughput phenotypic tools to obtain agronomically relevant indices in association mapping opens up the opportunity to use these indices to complement or replace other more laborious measurements in breeding programs. The GWAS results reported herein show marker–trait associations for ChlF and HSIs related to molecular function, cellular components, and biological processes. These results contribute to a better understanding of the genetic dissection of the ChlF and HS indices evaluated, which is directly or indirectly related to physiological processes in maize. Candidate gene analysis revealed a number of gene models across eight maize chromosomes. Among them, some are related to photosynthetic processes and plant stress responses. Our results corroborate the use of HS imaging in the context of maize breeding. Further research is needed to improve our understanding of biophysical modeling to develop spectral plant traits specific to drought tolerance.

## Figures and Tables

**Table 1:** Summary statistics for nine ChlF and HS indices indicating the overall mean of 165 maize inbred lines, standard deviation (SD), minimum and maximum values, and coefficient of variation (CV). The treatment/measurement combination in which significant genome-wide associations were detected is marked in bold.

Index	Treatment	Measurement	Mean	SD ^1^	Min	Max	CV
**ChlF indices**							
Phi_Po	Control	**1**	**0.81**	**0.02**	**0.69**	**0.84**	**2.30**
		2	0.79	0.02	0.69	0.84	2.93
		3	0.77	0.03	0.60	0.84	4.52
	Water Withholding	1	0.81	0.02	0.67	0.85	2.30
		2	0.77	0.04	0.57	1.21	5.53
		**3**	**0.62**	**0.18**	**0.00**	**1.22**	**29.12**
Psi_o	Control	1	0.60	0.05	0.25	0.72	8.46
		2	0.56	0.06	0.23	0.69	11.17
		3	0.52	0.09	0.13	0.70	18.24
	Water Withholding	1	0.59	0.06	0.30	0.71	9.59
		2	0.53	0.07	0.21	0.68	12.82
		**3**	**0.42**	**0.12**	**0.01**	**0.80**	**28.91**
**NIR indices**							
NDVI	Control	**1**	**0.41**	**0.03**	**0.12**	**0.50**	**8.02**
		2	0.40	0.03	0.19	0.47	7.35
		3	0.37	0.05	0.06	0.49	12.31
	Water Withholding	1	0.40	0.03	0.28	0.47	6.58
		2	0.37	0.04	0.13	0.49	10.87
		3	0.33	0.06	0.09	0.46	18.75
ZMI	Control	**1**	**1.78**	**0.14**	**1.13**	**2.17**	**8.10**
		2	1.69	0.15	1.14	2.12	9.14
		**3**	**1.56**	**0.19**	**0.89**	**2.04**	**12.14**
	Water Withholding	1	1.73	0.13	1.32	2.20	7.63
		2	1.63	0.19	1.02	2.12	11.52
		3	1.45	0.23	0.91	2.04	15.80
**UVIS indices**							
MCARI	Control	1	1718	479	137	5288	28
		2	1972	588	486	4694	30
		3	2489	915	330	6627	37
	Water Withholding	1	1844	461	518	4028	25
		**2**	**1974**	**760**	**57**	**5821**	**38**
		3	2148	893	124	6414	42
MCARI1	Control	**1**	**12** **,668**	**1081**	**3376**	**16** **,277**	**9**
		2	13,052	1199	3195	16,842	9
		**3**	**13** **,520**	**1723**	**0**	**19** **,422**	**13**
	Water Withholding	1	12,862	1128	4328	16,797	9
		**2**	**12** **,534**	**1721**	**0**	**17** **,700**	**14**
		3	11,025	2804	372	17,800	25
Greenness	Control	**1**	**1.44**	**0.07**	**1.14**	**1.79**	**4.92**
		2	1.48	0.09	1.20	1.83	5.74
		3	1.54	0.11	1.22	1.91	7.16
	Water Withholding	**1**	**1.45**	**0.07**	**1.21**	**1.73**	**4.52**
		2	1.46	0.10	1.10	1.87	6.68
		3	1.40	0.13	1.05	1.74	9.27
GM1	Control	**1**	**1.31**	**0.08**	**0.97**	**1.50**	**5.86**
		2	1.25	0.09	0.93	1.56	7.37
		3	1.17	0.11	0.75	1.47	9.31
	Water Withholding	1	1.29	0.08	0.95	1.48	5.83
		2	1.21	0.11	0.82	1.47	8.89
		3	1.18	0.11	0.82	1.49	9.70
GM2	Control	**1**	**1.65**	**0.09**	**1.17**	**1.86**	**5.56**
		**2**	**1.58**	**0.11**	**1.09**	**1.96**	**6.74**
		3	1.50	0.14	0.85	1.81	9.54
	Water Withholding	1	1.62	0.09	1.12	1.84	5.71
		2	1.52	0.14	0.90	1.85	9.04
		3	1.40	0.18	0.90	1.85	12.76

^1^ SD, standard deviation; Min, minimum value; Max, maximum value; CV, coefficient of variation.

**Table 2:** Single-nucleotide polymorphism (SNP) chromosomal positions on maize Chromosome 1 and candidate genes significantly associated with chlorophyll fluorescence and hyperspectral indices identified through the genome-wide association study. Gene names refer to Ensembl BioMart [34] and description and GO domains refer to MaizeGDB [35].

SNP	Position (bp)	Index-Measurement_Treatment ^1^	Gene	Description	GO Domain ^2^
SYN6732	8,131,266	Phi_Po-1_C	*Zm00001eb002960*	Aconitate hydratase (Aconitase) (EC 4.2.1.3)	MF; CC; BP
			*Zm00001eb002980*	2Fe-2S ferredoxin-type domain-containing protein	MF
			*Zm00001eb002990*	Scarecrow-like protein 6	MF; CC; BP
SYN11901	8,510,819	Phi_Po-1_C	*Zm00001eb003140*	Eisosome protein SEG2	BP
			*Zm00001eb003150*	MHD1 domain-containing protein	MF; CC; BP
			*Zm00001eb003170*	Proteasome component3; Protein NRT1/PTR FAMILY 5.2	MF; CC; BP
SYN5905	9,984,596	Phi_Po-1_C	*Zm00001eb003680*	ARM repeat superfamily protein	CC
			*Zm00001eb003690*	Tankyrase 1	MF; CC; BP
PZE-101206617	255,801,405	Phi_Po-1_C; ZMI-1_C	*Zm00001eb050200*	Gibberellin-regulated protein 1	CC
PZE-101206972	256,352,663	NDVI-1_C	*Zm00001eb050290 (Orphan251)*	Calmodulin binding protein; (Orphans transcription factor)	MF; CC; BP
PZE-101213333	263,494,471	NDVI-1_C	*Zm00001eb052020*	DUF4378 domain-containing protein	
SYN30053	297,383,893	NDVI-1_C	*Zm00001eb061650*	Protein TIFY (Jasmonate ZIM domain-containing protein)	CC; BP
			*Zm00001eb061670*	Rubredoxin-like superfamily protein; Rubredoxin-like domain-containing protein	MF; CC; BP
SYN30050	297,452,662	NDVI-1_C	*Zm00001eb061720*	Rubredoxin-like domain-containing protein	MF; CC; BP
			*Zm00001eb061740*	Phytocyanin domain-containing protein	MF; CC; BP
			*Zm00001eb061690*	Rubredoxin-like domain-containing protein	MF; CC; BP
			*Zm00001eb061730*	Rubredoxin-like domain-containing protein	
			*Zm00001eb061670*	Rubredoxin-like superfamily protein; Rubredoxin-like domain-containing protein	MF; CC; BP

^1^ Index-Measurement_Treatment: C—Control; W—Water Withholding. ^2^ Gene Ontology (GO) domain: MF—molecular function; CC—cellular component; BP—biological process.

**Table 3:** Single-nucleotide polymorphism (SNP) chromosomal positions on maize chromosomes 2, 3, and 4 and candidate genes significantly associated with chlorophyll fluorescence and hyperspectral indices identified through the genome-wide association study. Gene names refer to Ensembl BioMart [34] and description and GO domains refer to MaizeGDB [35].

SNP	Chr	Position (bp)	Index-Measurement_Treatment ^1^	Gene	Annotation	GO Domain ^2^
PZE-102083803	2	71,991,689	MCARI1-2_W	*Zm00001eb085350 (dnaJ_4)*	J domain-containing protein; DNAJ heat shock N-terminal domain-containing protein	MF; CC; BP
				*Zm00001eb085360 (NFD4_3)*	Major facilitator superfamily protein	CC; BP
SYN37731; SYN37721	3	13,322,314; 13,322,419	MCARI1-2_W	*Zm00001eb123160*	Autophagy-related protein 18c	MF; CC; BP
				*Zm00001eb123170*	RING-type domain-containing protein	MF; CC; BP
PZE-103025670	3	18,270,049	MCARI1-2_W	*Zm00001eb124560 (At2g39795_1)*	Mitochondrial glycoprotein	CC
				*Zm00001eb124570 (mcfB_1)*	Mitochondrial substrate carrier family protein; Mitochondrial substrate carrier family protein (Protein brittle-1)	MF; CC; BP
PZE-103031625	3	23,789,785	MCARI1-2_W	*Zm00001eb125530*	Mannose-6-phosphate isomerase (EC 5.3.1.8)	MF; CC; BP
SYN571; SYN572	3	29,970,155; 29,970,561	MCARI1-2_W	*Zm00001eb126630*	Uncharacterized protein	CC
PZE-103145413	3	200,419,345	MCARI1-3_C	*Zm00001eb152450*	Aspartate aminotransferase (EC 2.6.1.1)	MF; CC; BP
				*Zm00001eb152460*	Rop guanine nucleotide exchange factor 9; PRONE domain-containing protein	MF; CC; BP
PZE-103162732	3	213,380,441	Psi_o-3_W	*Zm00001eb156520 (RAX3_0)*	Transcription factor MYB36	MF
PZE-104005660	4	1,515,154	MCARI-2_W	*Zm00001eb164580*	protein ALTERED XYLOGLUCAN 4	-
PZE-104080257	4	154,445,958	MCARI-2_W	*Zm00001eb186100*	STI1 domain-containing protein; Hsp70-Hsp90 organizing protein 3	MF; CC; BP
				*Zm00001eb186110*	Uncharacterized protein	-
PZE-104137089	4	224,139,502	G-1_W	*Zm00001eb202870*	Protein DETOXIFICATION (Multidrug and toxic compound extrusion protein)	MF; CC; BP

^1^ Index-Measurement_Treatment: C—Control; W—Water Withholding. ^2^ Gene Ontology (GO) domain: MF—molecular function; CC—cellular component; BP—biological process.

**Table 4:** Single-nucleotide polymorphism (SNP) chromosomal positions on maize chromosomes 5 and 6 and candidate genes significantly associated with chlorophyll fluorescence and hyperspectral indices identified through the genome-wide association study. Gene names refer to Ensembl BioMart [34] and description and GO domains refer to MaizeGDB [35].

SNP	Chr	Position (bp)	Index-Measurement_Treatment ^1^	Gene	Annotation	GO Domain ^2^
PZE-105011679	5	5,105,787	Phi_Po-3_W	*Zm00001eb213320*	IQ-domain 5	MF; CC; BP
				*Zm00001eb213330*	Expp1 protein	MF; CC; BP
				*Zm00001eb213350*	t-SNARE coiled-coil homology domain-containing protein	CC; BP
SYN14962	5	6,920,822	Phi_Po-3_W	*Zm00001eb214310*	Protein arginine N-methyltransferase 2 (Type IV protein arginine N-methyltransferase); RMT2 domain-containing protein	CC; BP
				*Zm00001eb214320 (Orphan326)*	protein-serine/threonine phosphatase (EC 3.1.3.16)	MF; BP
				*Zm00001eb214330*	SWIb domain-containing protein	CC
				*Zm00001eb214350*	Gibberellin-regulated protein 10	CC; BP
				*Zm00001eb214360*	aspartate-semialdehyde dehydrogenase (EC 1.2.1.11)	MF; CC; BP
				*Zm00001eb214370*	TOG domain-containing protein	MF
				*Zm00001eb214380*	TIP41-like family protein	CC; BP
SYN26542	6	35,091,443	MCARI1-3_C	*Zm00001eb265310*	Protein kinase domain-containing protein (wakl32—wall associated kinase like32)	MF; CC; BP
SYN11972	6	88,729,282	GM2-2_C	*Zm00001eb271480*	Caffeoyl-CoA 3-O-methyltransferase 1	MF; CC; BP
SYN4313; SYN4314	6	165,501,556; 165,501,601	Phi_Po-1_C; GM1-1_C; GM2-1_C	*Zm00001eb290700*	Protein kinase domain-containing protein	MF; CC; BP
				*Zm00001eb290720*	Protein kinase domain-containing protein	MF; CC; BP
				*Zm00001eb290730*	Thioredoxin domain-containing protein	MF; CC; BP
				*Zm00001eb290740*	Secreted protein	-
SYN4302	6	165,504,772	Phi_Po-1_C	see *SYN4313*; *SYN4314*		

^1^ Index-Measurement_Treatment: C—Control; W—Water Withholding. ^2^ Gene Ontology (GO) domain: MF—molecular function; CC—cellular component; BP—biological process.

**Table 5:** Single-nucleotide polymorphism (SNP) chromosomal positions on maize chromosomes 9 and 10 and candidate genes significantly associated with chlorophyll fluorescence and hyperspectral indices identified through the genome-wide association study. Gene names refer to Ensembl BioMart [34] and description and GO domains refer to MaizeGDB [35].

SNP	Chr	Position (bp)	Index-Measurement_Treatment ^1^	Gene	Annotation	GO Domain ^2^
PZE-109031829	9	38,308,890	ZMI-3_C; MCARI-2_W	*Zm00001eb380540*	Protein kinase domain-containing protein	MF; BP
				*Zm00001eb380550*	SMAD/FHA domain-containing protein	MF; CC; BP
				*Zm00001eb380560*	Myb/SANT-like domain-containing protein	-
PZE-109060136	9	102,752,415	G-1_W	*Zm00001eb387560 (abhd17c_5)*	Alpha/beta-Hydrolases superfamily protein; Serine aminopeptidase S33 domain-containing protein	MF; CC; BP
				*Zm00001eb387550*	Uncharacterized protein	CC
				*Zm00001eb387540*	Intron maturase type II family protein	MF; CC; BP
PZE-109091063	9	138,752,843	G-1_W	*Zm00001eb395380*	GRAB2 protein	MF; BP
PZE-109108465	9	149,818,933	Phi_Po-1_C; G-1_C; GM2-2_C; MCARI1-1_C; NDVI-1_C	*Zm00001eb399040 (SMD3B_0)*	Small nuclear ribonucleoprotein Sm D3 (Sm-D3) (snRNP core protein D3)	MF; CC; BP
PZE-110000531	10	1,417,727	Phi_Po-3_W	*Zm00001eb405060 (NH5.1_1)*	Regulatory protein NPR5; BTB domain-containing protein	MF; CC; BP
				*Zm00001eb405080*	Scarecrow-like protein 3	-
				*Zm00001eb405110*	Scarecrow-like protein 3	MF; CC; BP
				*Zm00001eb405120*	Uncharacterized protein	CC

^1^ Index-Measurement_Treatment: C—Control; W—Water Withholding. ^2^ Gene Ontology (GO) domain: MF—molecular function; CC—cellular component; BP—biological process.

## Data Availability

Data available are from the authors upon request.
